# Ongoing challenges in the management of malaria

**DOI:** 10.1186/1475-2875-8-S1-S2

**Published:** 2009-10-12

**Authors:** Gilbert Kokwaro

**Affiliations:** 1 Consortium for National Health Research (CNHR), Nairobi, Kenya

## Abstract

This article gives an overview of some of the ongoing challenges that are faced in the prevention, diagnosis and treatment of malaria.

Malaria causes approximately 881,000 deaths every year, with nine out of ten deaths occurring in sub-Saharan Africa. In addition to the human burden of malaria, the economic burden is vast. It is thought to cost African countries more than US$12 billion every year in direct losses.

However, great progress in malaria control has been made in some highly endemic countries. Vector control is assuming a new importance with the significant reductions in malaria burden achieved using combined malaria control interventions in countries such as Zanzibar, Zambia and Rwanda. The proportion of patients treated for malaria who have a confirmed diagnosis is low in Africa compared with other regions of the world, with the result that anti-malarials could be used to treat patients without malaria, especially in areas where progress has been made in reducing the malaria burden and malaria epidemiology is changing. Inappropriate administration of anti-malarials could contribute to the spread of resistance and incurs unnecessary costs.

Parasite resistance to almost all commonly used anti-malarials has been observed in the most lethal parasite species, *Plasmodium falciparum*. This has presented a major barrier to successful disease management in malaria-endemic areas.

ACT (artemisinin-based combination therapy) has made a significant contribution to malaria control and to reducing disease transmission through reducing gametocyte carriage. Administering ACT to infants and small children can be difficult and time consuming. Specially formulating anti-malarials for this vulnerable population is vital to ease administration and help ensure that an accurate dose is received.

Education of healthworkers and communities about malaria prevention, diagnosis and treatment is a vital component of effective case management, especially as diagnostic policies change.

Preventing resistance emerging to both ACT and insecticides used in vector control remains an ongoing challenge in an era of changing malaria epidemiology.

## Background

Malaria is one of the world's most deadly diseases. Even though it is highly preventable and treatable, it causes approximately 881,000 deaths every year [1], with nine out of ten deaths occurring in sub-Saharan Africa, and 85% of malaria-related deaths in children under five years of age. This is the equivalent of a child dying of malaria in Africa every 30 seconds [2]. The most serious forms of the disease are caused by the parasite *Plasmodium falciparum*; malaria caused by *Plasmodium vivax*, *Plasmodium ovale *and *Plasmodium malariae *results in milder disease in humans that is not generally fatal. In 2008, 109 countries were reported to be endemic for malaria, with 45 of the countries within the African region [1]. There were an estimated 247 million episodes of malaria in 2006, with 86% of cases reported in African countries [3].

## The burden of malaria

Effective control and treatment of malaria presents enormous logistical challenges. The key to addressing the challenge of reducing the burden of malaria is an integrated approach that combines preventative measures, such as long-lasting insecticide-treated bed nets (LLINs) and indoor residual spraying (IRS), with improved access to effective anti-malarial drugs. However, malaria is a disease that stems from and causes poverty, and many at-risk populations live in extremely destitute, remote areas. Poor, rural families are the least likely to have access to these preventative measures that are fundamental to malaria control, and may live kilometres from the nearest healthcare facility. They are also less able to afford treatment once infection has occurred [4].

In addition to the human cost of malaria, the economic burden of the disease is vast. It is estimated that malaria costs African countries more than US$12 billion every year in direct losses [3], even though the disease could be controlled for a fraction of that sum. For Nigeria alone the direct loss to the economy is estimated at GBP530 million annually [3]. Up to 40% of African health budgets are spent on malaria each year [5], and on average, a malaria-stricken family loses a quarter of its income through loss of earnings and the cost of treating and preventing the disease [2]. Malaria causes an average loss of 1.3% of economic growth per year in Africa [3].

## The growing prominence of vector control

The success of malaria control measures in some highly-endemic countries, such as Rwanda, Zambia, Zanzibar, Sao Tome & Principe, The Gambia and Kenya has led to reductions in deaths from malaria of 50% or more [1]. In these countries, the widespread use of LLINs and IRS, together with treatment of uncomplicated *Plasmodium falciparum *malaria with artemisinin-based combination therapy (ACT), have all contributed to the reported reductions in malaria. Another article in this supplement describes the impact of combined malaria control interventions on disease burden [6].

These developments have led to an increased focus on vector control. The historic successful eradication of malaria in various parts of the world was achieved primarily by vector control, indicating that renewed efforts in this field, other than the current insecticide-based strategies, should be considered a central aspect of any malaria eradication strategy.

In the Roll Back Malaria (RBM) programme of the World Health Organization (WHO), vector control is based mainly on the continuation and upscaling of insecticide-based strategies. However, given the increasing prevalence of mosquito resistance against the currently used chemicals, the need for development of new insecticides has high priority. A multitude of novel malaria vector-control tools has been developed in recent years, and several of these are at an advanced stage, nearing broad-scale implementation. Recently, successes were reported in Kenya with *Bacillus thuringiensis israelensis *(Bti) for larval control [7]. Novel tools for the control of the adult mosquito include entomopathogenic fungi, insect-pathogenic viruses, the introduction of genetically engineered mosquitoes and the sterile insect technique (SIT) [8]. Field-based trials in which the impact of these vector-control tools on public health is properly measured are needed before they can be adopted to complement the malaria control tools currently in use.

## Improving malaria diagnosis

Accurate diagnosis is a vital part of good malaria case management and is becoming increasingly important as the need for presumptive treatment of fever declines along with malaria burden in many areas. The proportion of people treated for malaria who have a confirmed diagnosis is low in the African Region compared with other regions of the world [1], with the result that anti-malarials could be used to treat patients without malaria. Biological diagnostic methods such as rapid diagnostic tests (RDTs) are easy to use, give fast results and are increasingly affordable. As RDTs become more widely available, confirmation of malaria prior to treatment will become the standard procedure. The introduction of RDTs at the community level needs to be carefully planned, to include transport and storage considerations, local sensitivity testing and establishment of a comprehensive quality assessment/control system. Overcoming the tendency of some prescribers to treat with ACT despite a negative test result poses a significant challenge; training of end users should include how to manage positive and negative test results, and should also emphasize that a negative result is valid. There are currently no international guidelines or treatment algorithms for the management of negative tests results. Adequate training, supervision and follow-up are essential to achieving a change in perceptions and practice.

New WHO guidelines, due for publication in 2009, are expected to recommend that anti-malarials should not be given to febrile patients unless the parasitological presence of malaria has been confirmed by laboratory or rapid diagnostic testing. This reduces the unnecessary administration of these drugs and may also help to combat the spread of resistance by ensuring that only malaria patients receive this treatment. It will also mean that patients who present with a fever but do not have malaria are more likely to be given an appropriate treatment more rapidly.

This revised approach to malaria diagnosis may also have an impact on patients' perceptions of anti-malarial therapies. Patients will have greater confidence in a therapy if it is shown to work quickly and effectively. If an ACT is administered for a non-malarial infection, the patient will not feel satisfied as the drug will not improve their symptoms. The patient may, therefore, be less likely to adhere to an ACT regimen in the future if an actual diagnosis of malaria is made, due to their previously negative experience with that drug. Thus, it is vital that the initial diagnosis is correct to encourage future compliance.

## Drug resistance as a barrier to malaria control

The development and spread of parasite resistance to certain anti-malarial agents has presented a major barrier to successful disease management in malaria-endemic areas, and has probably contributed to the resurgence of infection and the increase in malaria-related deaths in recent years [9]. Resistance to almost all commonly used anti-malarials, notably chloroquine and sulphadoxine-pyrimethamine, but also amodiaquine, mefloquine, and quinine, has been observed in the most lethal parasite species, *P. falciparum *[9]. The problem of resistance exists in much of Africa and Southeast Asia [10-12]; for example, treatment failure rates of around 70-80% have been reported for chloroquine [10], which was formerly the cheapest and most widely available anti-malarial drug. There is also increasing drug resistance to amodiaquine in large parts of East Africa, potentially rendering the combination of artesunate and amodiaquine less effective [11].

Artemisinin derivatives are the only class of anti-malarial agents to which *P. falciparum *resistance has not been reported in Africa [9], and as a result the WHO called for the use of artemisinin in combination therapies in September 2005 [4,5]. In January 2006, WHO called for termination of the distribution and sale of artemisinin monotherapies by pharmaceutical companies [9]. Unfortunately, many companies are yet to comply, creating a further barrier to disease control. In addition, substandard drugs and counterfeits pose a major problem. Anti-malarial drugs are widely available outside the public health services; for example, from pharmacies, shops, private practitioners and other outlets. It is thought that up to 35% of all anti-malarial treatments sold in Africa are sub-standard [13].

The half-life of anti-malarials is believed to be an important factor in the development and spread of resistance [14,15]. It has been suggested that compounds with a longer half-life have a greater propensity to become ineffective due to parasite resistance. For a drug that has a half-life of weeks or months (e.g. mefloquine, piperaquine or chloroquine), the slow elimination from the host's blood enables parasites to be exposed to residual drug concentrations. Malaria parasites will not be exposed to partially effective drug concentrations if the drugs are eliminated during the two-day life cycle of the asexual parasite [14]. However, it has also been proposed that a longer half-life offers protection against re-infection for a longer period of time - this effect is known as "post-treatment prophylaxis", and represents the period of time after an anti-malarial treatment dose during which reinfection is suppressed [16,17]. Thus, what benefits the individual patient may be detrimental to society if there is a greater risk of resistance developing as more parasites are exposed to sub-therapeutic drug levels.

Using anti-malarials that have evolved from similar basic chemical compounds can lead to an increase in the development of resistance. For example, the relatively high rate of treatment failure reported with the ACT dihydroartemisinin-piperaquine against *P. falciparum *may be attributed to cross-resistance between chloroquine and piperaquine [18]. This may reduce the usefulness of new therapies prior to large-scale deployment if they are derivatives of currently used drugs to which resistance has already been established.

## Reducing malaria transmission

### Using effective anti-malarials

Rapid treatment of malaria cases with a treatment that quickly reduces the parasite load and is also strongly gametocidal can help reduce the rate of malaria transmission.

Artemisinin derivatives are extremely potent anti-malarials with a rapid onset of action, and when administered in combination with anti-malarial drugs with slower elimination rates (e.g. lumefantrine), short courses of treatment (three days) have proved to be highly effective [9]. The less effective single-drug treatments increase the chance of parasites evolving and becoming resistant to the treatment; combining anti-malarial drugs with independent modes of action can impede the development of resistance to each individual component of the combination. In the rare event that a mutant parasite resistant to one of the drugs arises *de novo *during the course of an infection, the parasite will be killed by the other drug in the combination [9].

Artemisinin derivatives can reduce parasite load by a factor of approximately 10,000 per asexual cycle [19], compared with a reduction of 100-fold to 1,000-fold per cycle with most other anti-malarial drugs [9]. They also have gametocidal properties, which could have an important effect on the incidence of malaria. Artemether can lower transmission of the infection by inhibiting gametocyte development, thus reducing the dissemination of resistant parasites. Evidence has shown a more rapid time to gametocyte clearance with artemether/lumefantrine (AL) than with other anti-malarial combinations [20].

**Figure 1:**
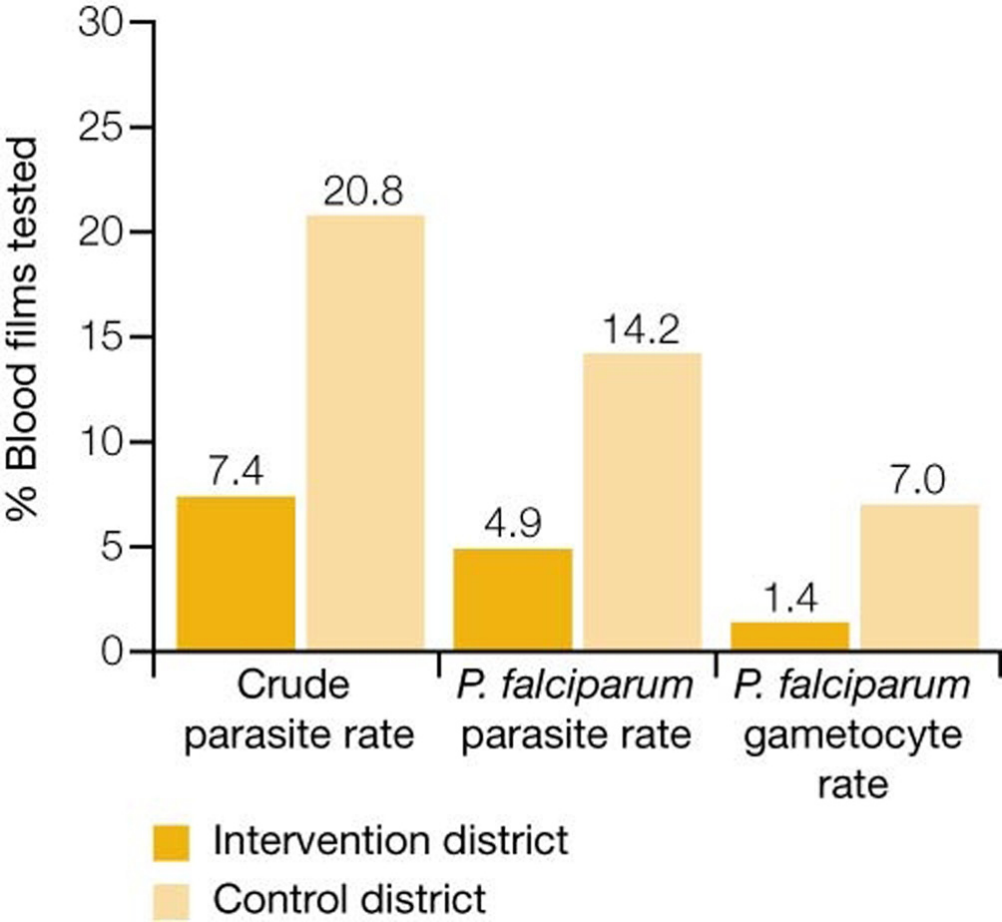
**Malaria parasite reservoir in the control and intervention districts of the Tigray study region**. Malaria parasite reservoir was 3-fold lower in the intervention district during 2005 high-transmission season.

A study in the Tigray region of Ethiopia compared community deployment of AL with traditional health facility-based care, finding that even during a major malaria epidemic, malaria transmission was decreased. The control district (health facility based-care) had a three-fold higher crude parasite rate (all species and stages), a two-fold higher crude asexual parasite rate (*P. vivax *and *P. falciparum*) and an approximately threefold higher *P. falciparum *rate (both asexual and gametocyte stages) than the intervention (community deployment) district (Figure 1). This indicates that the intervention had an effect, as all other vector control activities were comparable, with even better coverage in the control district [21].

### Treatment of asymptomatic carriers

Asymptomatic carriers of *P. falciparum *are likely to be essential for maintaining the cycle of infection in areas of high malaria transmission. Treatment of asymptomatic carriers could help reduce disease transmission by depleting the reservoir of parasites available for infection of mosquitoes. In comparison with non-artemisinin regimens, treatment with artemisinin derivatives has been shown to result in lower gametocyte carriage rates [22,23], and reduced infectivity of treated individuals [24].

In a study in The Gambia, children treated with AL were significantly less likely to carry gametocytes within the four weeks following treatment than those receiving chloroquine and sulphadoxine-pyrimethamine (p < 0.0001) [24]. In addition, children who received AL harboured gametocytes for shorter periods following treatment (p < 0.0001) and were less infective to mosquitoes at day 7 (p < 0.001) [24].

However, before the treatment of asymptomatic carriers can be considered as a new tool in the efforts to eliminate malaria, evidence of the proof of concept will need to be generated from suitably powered trials.

### Role of bed nets

Widespread deployment of LLINs has proved a very successful contribution to malaria control strategy. In Rwanda, Ethiopia, and Zanzibar, the mass distribution of insecticide-treated bed nets and nationwide adoption of ACT resulted in substantial declines in malaria-related deaths [25,26]. However, the impact of LLINs distribution can be lost if vulnerable populations do not use the nets or re-purpose them. A study in western Kenya found that 30% of bed net recipients did not adhere to net use [27,28]. A study of fishing villages on Lake Victoria in Kenya reported bed nets being used for fishing and drying fish, with reasons given that the nets were inexpensive or free and allowed the fish to dry very quickly [29].

In addition, the effectiveness of bed nets in helping to prevent disease transmission is dependent on their integrity and longevity. A study in Ghana revealed that only 14.9% of 255 nets collected for analysis 38 months after distribution had retained their full insecticidal strength. Of 50 nets examined for holes, more than 40% contained holes of more than 0.5 cm diameter, and 50% had seam failures [30].

## Meeting the need for a paediatric formulation

The provision of an appropriate formulation of ACT for infants and young children, who bear the greatest burden of malaria, presents a particular challenge. Administering anti-malarials to infants and small children can be difficult, stressful, and time consuming. Many malaria tablets need to be crushed and mixed with food or water to ease administration to young children, and the bitter taste can cause children to spit out the crushed tablets and, therefore, possibly not receive an optimal dose that will cure the malaria infection. Some existing paediatric drugs are presented as syrups or suspensions, which can be bulky to supply and store, and the stability and hygiene of the formulation cannot be guaranteed after opening. Accurate dosing of syrups may be difficult as it requires precise volume measurement in the field.

A new dispersible formulation of AL, specifically designed for children, has proved in clinical trials to be as safe and effective as the regular tablet formulation [31]. Dispersible AL tablets (Coartem^® ^Dispersible) rapidly disperse in a small amount of water to produce a sweet-tasting formulation for ease of administration to infants and children [32]. Accurate dosing is aided by Coartem^® ^Dispersible tablets (as with regular Coartem^®^tablets) being administered as a fixed dose, according to specific weight categories of the patients [32,33].

## Training, educating and sharing best practice

Educating healthcare workers and patients about the prevention and treatment of malaria is another challenge in the management of the disease. Novartis has an ongoing commitment to developing initiatives, such as educational materials and training courses for healthcare workers, and the communities they serve; examples include a malaria case management programme for nurses in Zambia, and the development of educational materials for healthcare workers and mothers/caregivers, which have been translated into several African languages and are distributed free of charge to countries on request.

One of the greatest difficulties in reducing the toll of malaria is reaching remote communities with poor transport systems, and achieving timely reordering to maintain supplies of ACT. The procurement of anti-malarial medicines through public health services increased sharply between 2001 and 2006, but access to treatment, especially ACT, was inadequate in all countries surveyed in 2006 [1]. Novartis is helping to address these challenges by hosting a series of biannual workshops in Africa, at which national malaria control programme (NMCP) managers can share information regarding best practice in their countries, including topics such as community awareness, healthcare worker training, stock management and distribution, and health impact measurement. Participants can also share expertise on how to forecast demand for AL, as well as discussing ordering and distribution systems, and routes for financing and procurement.

## Conclusion

Malaria continues to be a significant public health issue and a major hindrance to economic growth. Sustained control and management of malaria presents significant challenges. Effective interventions are available; these include widespread implementation of effective vector control measures such as LLINs and IRS, and prompt and effective treatment with ACT following accurate diagnosis. Reducing malaria transmission by reducing gametocyte carriage with effective drugs can be an important factor in highly endemic areas. Some endemic countries are forging a path in malaria control and prevention using combined interventions and have achieved significant reductions in malaria burden.

However, factors such as patient access to effective treatments and preventative measures, availability of training programmes and educational materials, and development and spread of resistance to certain anti-malarials are hindering progress.

Addressing the continuing challenges presented by malaria in the years ahead will require responsive strategies such as innovative vector control methods, widespread implementation of biological diagnosis prior to treatment with effective anti-malarials, and close monitoring of local malaria epidemiology to identify areas of resurgence. Tight control of the regulatory environment to ensure provision of high-quality drugs and appropriate dosing advice will be vital to promoting compliance and preserving effectiveness of ACT.

## Competing interests

The author would like to acknowledge that Novartis Pharma AG sponsored this supplement. However, the author does not work for, or represent in any way, Novartis Pharma AG.

## Authors' contributions

The author met International Committee of Medical Journal Editors criteria for authorship.
